# Zygosity‐Dependent Phenotypic Spectrum of *RELN*‐Related Disorders: 10 New Patients and Genotype–Phenotype Correlations Across 48 Kindreds

**DOI:** 10.1155/humu/9993570

**Published:** 2026-09-17

**Authors:** Sajjad Biglari, Halimeh Rezaei, Elnaz Asadollahzadeh, Mohammad Salimi Asl, Hamid Galehdari, Masoud Garshasbi, Emran Esmaeilzadeh, Raziyeh Khalesi, Payman Jamali, Farshid Parvini, Gholamreza Shariati, Niloofar Chamanrou, Reza Saligheh, Atefeh Sohanforooshan Moghaddam, Hamid Reza Khorram Khorshid, Sepideh Shohani, Samira Nafar, Atieh Eslahi, Mojtaba Lotfi, Behnoosh Bakhshoodeh, Fatemeh Hajizadeh, Saeed Abtahi, Reza Gharaei Jomehei, Hassan Vahidnezhad, Mehran Beiraghi Toosi, Morteza Heidari, Hanieh Mirzadeh, Majid Mojarrad, Mina Mohammadi Sarband, Ehsan Ghayoor Karimiani

**Affiliations:** ^1^ Farin Genetics Laboratory, Tehran, Iran; ^2^ Department of Genetics, Faculty of Biological Sciences and Technology, Shahid Ashrafi Esfahani University, Isfahan, Iran, ashrafi.ac.ir; ^3^ Multiple Sclerosis Research Center, Neuroscience Institute, Tehran University of Medical Sciences (TUMS), Tehran, Iran, tums.ac.ir; ^4^ Medical Genetics Department, Genetic Diagnosis Center, Colife Integrated Medical Laboratories, Tehran, Iran; ^5^ Medical Genetics Department, VestaLab Genetic Diagnosis Center, Tehran, Iran; ^6^ Department of Biology, Faculty of Science, Shahid Chamran University of Ahvaz, Ahvaz, Iran, scu.ac.ir; ^7^ Department of Medical Genetics, Faculty of Medical Sciences, Tarbiat Modares University, Tehran, Iran, modares.ac.ir; ^8^ Genetics Research Center, University of Social Welfare and Rehabilitation Science, Tehran, Iran; ^9^ Department of Medical Genetics, DeNA Laboratory, Tehran, Iran; ^10^ The Genetic Counseling Center, Shahroud Welfare Organization, Shahroud, Iran; ^11^ Department of Biology, Faculty of Basic Sciences, Semnan University, Semnan, Iran, semnan.ac.ir; ^12^ Department of Medical Genetics, School of Medicine, Ahvaz Jundishapur University of Medical Sciences, Ahvaz, Iran, ajums.ac.ir; ^13^ Narges Medical Genetics and Prenatal Diagnosis Laboratory, Ahvaz, Iran; ^14^ Genetic Counseling Center, State Welfare Organization, Qom, Iran; ^15^ Department of Medical Biotechnology, School of Allied Medical Sciences, Iran University of Medical Science, Tehran, Iran, iums.ac.ir; ^16^ Department of Molecular Biology and Anatomical Sciences, School of Medicine, Isfahan University of Medical Sciences, Isfahan, Iran, mui.ac.ir; ^17^ Department of Medical Genetics, Faculty of Medicine, Mashhad University of Medical Sciences, Mashhad, Iran, mums.ac.ir; ^18^ Clinical Research Development Unit, Akbar Hospital, Faculty of Medicine, Mashhad University of Medical Sciences, Mashhad, Iran, mums.ac.ir; ^19^ Department of Dermatology, Mashhad University of Medical Sciences, Mashhad, Iran, mums.ac.ir; ^20^ Faculty of Medicine, Torbat Heydariyeh University of Medical Sciences, Torbat Heydariyeh, Iran, thums.ac.ir; ^21^ Department of Pediatric Cardiology, MMSC, Islamic Azad University, Mashhad, Iran, tiau.ac.ir; ^22^ Faculty of Medicine, Islamic Azad University, Mashhad, Iran, tiau.ac.ir; ^23^ Department of Pediatrics, Perelman School of Medicine, University of Pennsylvania, Philadelphia, Pennsylvania, USA, upenn.edu; ^24^ Center for Applied Genomics, Children′s Hospital of Philadelphia, Philadelphia, Pennsylvania, USA; ^25^ Rare Pediatric Neurological Diseases Research Center, Mashhad University of Medical Sciences, Mashhad, Iran, mums.ac.ir; ^26^ Myelin Disorders Clinic, Department of Pediatric Neurology, Children′s Medical Center, Pediatrics Center of Excellence, Tehran University of Medical Sciences, Tehran, Iran, tums.ac.ir; ^27^ Department of Pediatrics, Imam Sajad Hospital, Iran University of Medical Science, Tehran, Iran, iums.ac.ir; ^28^ Genetic Foundation of Khorasan Razavi, Mashhad, Iran; ^29^ Genetic Counseling Center, State Welfare Organization, Tehran, Iran; ^30^ Department of Neuromuscular Disorders, UCL Queen Square Institute of Neurology, London, UK

**Keywords:** ACMG/AMP variant classification, autosomal dominant lateral temporal epilepsy, exome sequencing, genotype–phenotype correlation, lissencephaly with cerebellar hypoplasia, Reelin, *RELN*

## Abstract

Variants in *RELN* lead to a range of neurodevelopmental phenotypes, from autosomal recessive lissencephaly with cerebellar hypoplasia (LCH) (LIS2) to autosomal dominant focal epilepsies. We carried out exome sequencing (ES) on 10 affected individuals from eight unrelated Iranian families with consanguinity. Nine distinct variants were identified: Four are novel; four had been deposited in ClinVar without a reported affected individual; and the ninth, c.2015C>T, p.(Pro672Leu), had been reported only in heterozygous carriers with dominant epilepsy. In this study, we report the variant for the first time in the homozygous state, in a patient with the full recessive phenotype. The patients were pooled in a systematic review following PRISMA, updated to July 2026, with 28 papers, and all variants were reannotated against NM_005045.4, under a single ACMG/AMP framework. The primary cohort comprised 80 individuals from 48 kindreds (70 previously reported, 10 new) with 48 distinctive variants, of which 41 (85%) were pathogenic or likely pathogenic and five were variants of uncertain significance; two balanced rearrangements were not in either the sequence‐variant or copy‐number frameworks; and 16 individuals from nine kindreds with monoallelic candidate or susceptibility variants were analyzed separately. The age of onset was not continuous with no reports of onsets between 2.4 and 8 years. Zygosity did not account for this separation because monoallelic individuals occurred in both onset groups; in contrast, malformations of cortical development were documented in all 32 early‐onset individuals and in none of the 23 later onset individuals with evaluable neuroimaging. ES was the diagnostic method in 65% of individuals. Thus, zygosity contributes to the phenotypic severity in a broad spectrum of RELNopathies and supports carrier testing and reproductive counseling in consanguineous families.

## 1. Introduction

The Reelin gene (*RELN*; Mendelian Inheritance in Man [MIM] 600514) produces a large extracellular glycoprotein that acts as a critical factor for neuronal migration, brain development, and synaptic organization in both the developing and the mature brain [1, 2]. The binding of Reelin to the very‐low‐density lipoprotein receptor (VLDLR) and to apolipoprotein E receptor 2 (ApoER2) triggers activation of the intracellular adaptor disabled‐1 (DAB1), which directs migrating neurons to their proper laminar positions within the cerebral and cerebellar cortices [3]. Loss of Reelin signaling therefore prevents neurons from reaching their laminar positions, resulting in a range of neurodevelopmental phenotypes in humans whose severity reflects the extent of Reelin deficiency.

At the severe end of this spectrum is autosomal recessive (AR) lissencephaly with cerebellar hypoplasia (LCH) (lissencephaly 2, LIS2; MIM 257320), characterized by impaired neuronal migration during corticogenesis, resulting in a simplified gyral pattern, abnormal cortical thickness, and significant cerebellar underdevelopment [4, 5]. Affected children usually appear within the first few months of life with neonatal hypotonia, widespread developmental delays, and seizures. Over time, they also exhibit intellectual disability and cerebellar dysfunction. The developmental issues stem from the underlying malformation and occur regardless of the seizure severity. The human phenotype mirrors that of the reeler mouse, which lacks Reelin and displays similar cortical disorganization [6].

Research has identified *RELN* spectrum disorders to include autosomal dominant lateral temporal epilepsy (ADLTE) and other focal epilepsies [7]. In familial epilepsy disorders without cortical malformation, heterozygous *RELN* variants are recognized as another important genetic cause. In these families, heterozygous *RELN* variants, usually missense mutations that disrupt Reelin folding and secretion, are present without causing cortical malformation [8]. These findings indicate that pathogenic variants in *RELN* may lead to either structural brain abnormalities or localized epilepsy, depending on the variant type, zygosity, and molecular implications.

Although numerous individual cases have been documented, the distinction between genotype and phenotype is still unclear. Biallelic loss‐of‐function (LOF) variants eliminate the production or secretion of functional Reelin, leading to severe LCH [4, 5, 9, 10]. Monoallelic variants are linked to two presentations: de novo missense variants impairing Reelin secretion and sometimes acting dominantly‐negative on wild‐type protein, leading to cortical malformations like pachygyria and polymicrogyria [11, 12]; and inherited missense variants with reduced penetrance that contribute to ADLTE and similar focal epilepsies, occurring without detectable cortical malformations [7, 10, 13]. Rare monoallelic variants have also been identified as candidate or susceptibility alleles in autism spectrum disorder [14–17] and schizophrenia [18–20]. Dazzo et al. [10] suggested that various presentations could be due to a single zygosity‐dependent mechanism, and Di Donato et al. [11] later described a spectrum of *RELN*‐related neurodevelopmental abnormalities in a clinically identified cohort of malformations. That cohort was not assembled as a systematic review and did not include ADLTE or neuropsychiatric literature. As a result, the recessive malformation and monoallelic epilepsy and neuropsychiatric literature have not been combined and standardized within a single dataset.

We present 10 cases from eight consanguineous Iranian families with biallelic *RELN* variants and the LCH phenotype, including the first homozygous patient with the c.2015C>T, p.(Pro672Leu) variant, previously reported only in dominant epilepsy families. We apply a primary cohort of 80 individuals from 48 independent kindreds to reannotate each variant against NM_005045.4 in a single American College of Medical Genetics and Genomics/Association for Molecular Pathology (ACMG/AMP) framework. We are aimed at exploring whether the phenotypic range of *RELN*‐related disorders (RELNopathies) is stratified by zygosity and at providing a uniformly classified reference dataset to aid in the interpretation of *RELN* variants in a diagnostic setting.

## 2. Materials and Methods

### 2.1. Compliance With Ethical Standards

Shahid Chamran University of Ahvaz, Iran′s Ethics Board in the Genetics Department approved this study (code: EE/99.3.02.39641/scu.ac.ir). The research involving human participants followed all relevant national laws and institutional policies, adhered to the Helsinki Declaration principles, and received approval from the authors′ institutional review board or an equivalent committee.

### 2.2. Subject Recruitment and DNA Extraction

**Figure 1:**
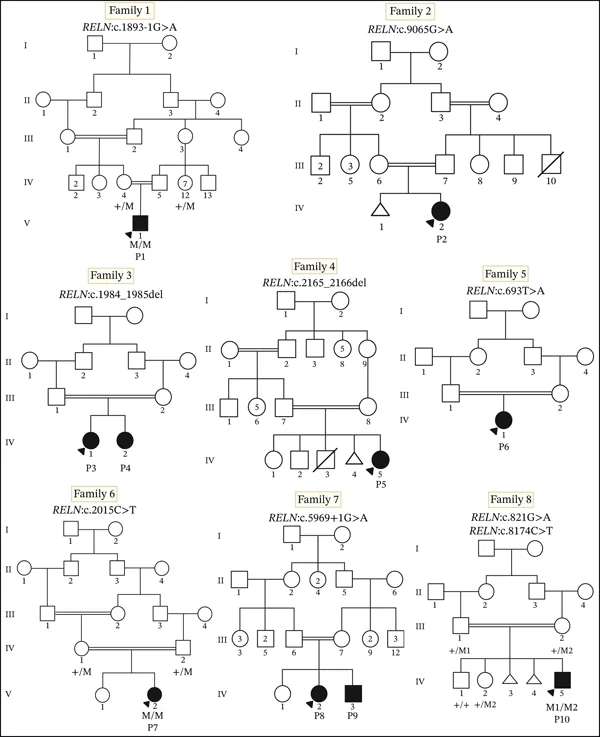
Pedigree of the eight studied families with the *RELN* variants. Genotypes are shown beneath tested individuals: “+” denotes the wild‐type allele and “M” the familial *RELN* variant. In Family 8, two different variants segregate: M1 = c.821G > A, p.(Arg274His), inherited from the heterozygous father, and M2 = c.8174C > T, p.(Ser2725Phe), inherited from the heterozygous mother; the proband (P10) carries M1/M2 in trans. Individuals without a genotype beneath the symbol were not tested.

In this study, eight unrelated Iranian families were included (Figure 1). Genetic counseling was performed to obtain medical histories and pedigree development (BioRender.com). After written informed consent was obtained, peripheral blood was collected and genomic DNA was extracted using the AddPrep Genomic DNA Extraction Kit (AddBio, Korea). The quality and quantity of DNA were checked by agarose gel analysis and Nanodrop 2000 (Thermo Fisher Scientific, United States) prior to exome sequencing (ES).

### 2.3. ES

A sample of 300 ng of genomic DNA was sent to Macrogen in South Korea. DNA was fragmented and enriched using the Agilent SureSelect Human All Exon V7 kit. The Illumina NovaSeq 6000 platform produced sequencing data from libraries that achieved more than 90x average coverage and 150x mean depth and delivered greater than 92% coverage of targeted bases. ES was conducted in single‐sample (singleton) mode on the affected proband of each of the eight families; no relative underwent ES. Candidate variants were validated in the proband by Sanger sequencing and segregation was determined by Sanger sequencing of all available parents and siblings. By Sanger sequencing, the familial variant was not tested in the two affected nonprobands (P4 and P9).

### 2.4. Data Analysis

Raw sequencing reads were converted into FASTQ files and aligned to the human reference genome (hg38) using the Burrows–Wheeler Aligner (BWA) (http://bio-bwa.sourceforge.net/). Base quality recalibration and variant calling were conducted with the Genome Analysis Toolkit (GATK) (https://www.broadinstitute.org/gatk/). All procedures followed the methods detailed in our previous publications [21–24].

### 2.5. Variant Classification

We used ACMG/AMP standards and guidelines [25], to classify variants, incorporating ClinGen Sequence Variant Interpretation (SVI) recommendations for specific criteria. PVS1 was assigned based on the SVI decision tree for predicted LOF variants [26]. PM2 was used at supporting strength following the SVI recommendation for the absence/rarity criterion with a maximum credible allele frequency of 0.001 for *RELN*. PM3 was applied at a lower weight for homozygous genotypes ascertained in consanguineous pedigrees in which homozygosity is predicted by descent and was not used in situations where the trans variant was itself unclassified. PP1 strength was assigned based on the number of informative meioses or if a cosegregation logarithm of the odds (LOD) score was used, based on established quantitative thresholds [27, 28]. PP4 was used with moderate strength for LCH, a phenotype that in practice is due to *RELN* or *VLDLR* and that has a high molecular diagnostic yield [28–30], provided that no other candidate was found in the ES. PP5 was not used, following the SVI recommendation not to apply PP5 [31].

Automated classifications from VarSome Premium served only as initial references and were manually verified for each variant, since automated tools lack access to case‐level zygosity and family segregation data, and thus do not incorporate PM3 or PP1 criteria. All variants, whether from this cohort or identified through the systematic review, were annotated against a single transcript, NM_005045.4, following HGVS nomenclature, and evaluated using gnomAD v4.1.0. The criteria and their assigned strengths for each variant are detailed individually in Table S6.

### 2.6. Bioinformatics Tools

Population allele frequencies were retrieved from the Genome Aggregation Database (gnomAD v4.1.0). In silico prediction of variant pathogenicity was assessed using multiple algorithms, including FATHMM, FATHMM‐MKL, LIST‐S2, M‐CAP, MutationAssessor, MutPred, PROVEAN, SIFT and SIFT4G, MutationTaster, BayesDel (addAF/noAF), MetaLR, MetaRNN, REVEL, DEOGEN2, and CADD (v1.7). The analysis used Ensemble consensus and conservation methods to improve the interpretation of variants.

### 2.7. Protein Modeling

The PyMOL Molecular Graphics System Version 2.5.7 performed structural modeling of the human Reelin protein (UniProt P78509). We displayed protein domains together with variant positions to assess possible structural changes.

### 2.8. Systematic Review

The systematic review was conducted and reported following the PRISMA 2020 (Preferred Reporting Items for Systematic Reviews and Meta‐Analyses) guidelines [32].

### 2.9. Eligibility Criteria

Eligible individuals were categorized into one of two datasets through three predefined routes: Route 1: Patients having a biallelic *RELN* genotype and a concordant neurological or cortical malformation phenotype were assigned to the primary quantitative cohort. Route 2: Individuals with a monoallelic variant reported as causal by the original authors in the setting of a malformation of cortical development or a familial focal epilepsy, supported by segregation, de novo, or functional data, were included in the primary cohort. Route 3: Individuals identified in association studies of autism spectrum disorder or schizophrenia were included in the primary cohort only if the variant was classified as pathogenic or likely pathogenic upon reannotation, either in the sequence‐variant framework based on de novo status or published functional data, or, for two intragenic deletions deleting coding exons of *RELN*, in the ClinGen/ACMG copy‐number standards; all other individuals were kept in the candidate/susceptibility dataset. Routes 1 and 2, which comprise 74 of the 80 individuals in the primary cohort, are independent of variant classification, whereas Route 3, which comprises the remaining six, is not. The classification distribution is then shown for the full primary cohort, and as a sensitivity analysis for the subset assigned by routes 1 and 2 only.

### 2.10. Search Strategy

The database search was first performed on September 3, 2023, and updated through July 31, 2026. The electronic bibliographic databases Scopus, Embase, MEDLINE, Web of Science, and Google Scholar were searched systematically using previously described approaches [33, 34]. Keywords associated with the *RELN* gene served as search criteria (see Table S1). Our search had no publication date restrictions. English was the only language permitted.

We imported all retrieved articles into EndNote and Excel for screening, removing duplicate papers and patients. For example, Michelucci et al. [7] report the same seven families and 28 individuals previously described by Dazzo et al. [10] without independent genotyping; it was cited for clinical detail but treated as a duplicate rather than an independent study. To ensure maximum accuracy, we also examined the “Reference” sections of relevant articles to identify additional *RELN* cases [33].

### 2.11. Selection Process

Two researchers (S.B. and A.S.M.) independently screened titles, abstracts, and full texts. Disagreements were settled through discussion, and senior researchers (S.B. and E.G.K.) were consulted if consensus could not be reached.

### 2.12. Data Collection Process

Out of the 28 eligible studies, 21 contributed data to the primary quantitative cohort, whereas seven were solely related to the susceptibility dataset. From these, we extracted information on (i) causative gene, (ii) mode of inheritance, (iii) neurological features, (iv) number of patients, (v) age of onset, and (vi) ethnicity. The gene symbols and protein names were selected based on the recommendations from the Human Genome Organization′s Gene Nomenclature Committee (HUGO).

### 2.13. Quality Assessment

Study quality was assessed using the Joanna Briggs Institute critical appraisal tools for case reports, case series, and cross‐sectional studies (http://joannabriggs.org). It was graded as good, fair, or poor based on whether all, some, or key criteria were satisfied (see Table S2).

### 2.14. Statistical Analysis

Descriptive statistics are presented as mean ± standard deviation (SD) for normally distributed variables and as median and interquartile range (IQR) for other variables. Age‐of‐onset data were summarized separately for each mode of inheritance. If only the range of onset at the family level was available, the midpoint was used to describe the onset. Values less than 1 year were recorded as 0.5 years. All combined analyses of demographics, clinical features, age of onset, inheritance patterns, diagnostic methods, and ACMG classifications excluded susceptibility cases.

## 3. Results

### 3.1. Clinical Findings

Ten patients from eight consanguineous families were found to harbor biallelic *RELN* variants (Figure 1). All 10 cases had clinical symptoms recorded with terms from the Human Phenotype Ontology (HPO) and are summarized in Table 1. The patients were seven women and three men from consanguineous families.

**Table 1:** Overview of core clinical features in *RELN*‐deficient patients (NM_005045.4).

Manifestations	P1 (F1)	P2 (F2)	P3 (F3)	P4 (F3)	P5 (F4)	P6 (F5)	P7 (F6)	P8 (F7)	P9 (F7)	P10 (F8)	
Ethnicity	Iranian	Iranian	Iranian	Iranian	Iranian	Iranian	Iranian	Iranian	Iranian	Iranian	
Sex	Male	Female	Female	Female	Female	Female	Female	Female	Male	Male	
Variants	c.1893‐1G>A	c.9065G>A	c.1984_1985del	c.1984_1985del	c.2165_2166del	c.693T>A	c.2015C>T	c.5969+1G>A	c.5969+1G>A	c.821G>A	c.8174C>T
Protein	—	p.(Trp3022Ter)	p.(Met662ValfsTer4)	p.(Met662ValfsTer4)	p.(Ser722LeufsTer3)	p.(Cys231Ter)	p.(Pro672Leu)	—	—	p.(Arg274His)	p.(Ser2725Phe)
Type of variants	Splicing	Stop gain	Frameshift	Frameshift	Frameshift	Stop gain	Missense	Splicing	Splicing	Missense	
Exon/intron	Intron 15	Exon 56	Exon 16	Exon 16	Exon 18	Exon 7	Exon 17	Intron 39	Intron 39	Exon 9	Exon 51
ACMG scoring	PVS1, PM2_S, PP4	PVS1, PM2_S, PP4	PVS1, PM2_S, PP1, PP4	PVS1, PM2_S, PP1, PP4	PVS1, PM2_S, PP4	PVS1, PM2_S, PP4	PM2_S, PP3_M, PP4_M, PM3_S,	PVS1, PM2_S, PP1, PP4	PVS1, PM2_S, PP1, PP4	PM2_S, PP3_S, PP4_S	PM2_S, PP3_S, PP4_S
ACMG prediction	P	P	P	P	P	P	LP	P	P	VUS	VUS
Zygosity	Hom	Hom	Hom	Hom	Hom	Hom	Hom	Hom	Hom	Compound Het	
Consanguinity	+	+	+	+	+	+	+	+	+	+	
Age (years)	7	2	11	8	2.1	5.5	2	19	10	2.5	
Age of onset (years)	2.4	0	0.1	2	0	2	0	0	0	0	
Initial symptoms	DD	DD	Seizures	Seizures	DD and glaucoma	—	DD	DD	DD	DD	
Seizures	+	+	+	+	+	−	−	+	+	+	
Hypotonia	+	+	+	+	+	+	+	+	+	+	
Lissencephaly	+	+	+	+	+	+	+	+	+	+	
Microcephaly	−	+	+	+	+	+	+	+	+	+	
Developmental delay	+	+	+	+	+	+	+	+	+	+	
Poor speech	+	+	+	+	+	+	+	+	+	+	
Poor growth	+	+	+	+	+	−	+	−	−	+	

*Note:* DD, developmental delay; M, moderate (ACMG criterion strength); S, supporting (ACMG criterion strength).

### 3.2. Molecular and In Silico Findings

We performed ES to identify the underlying genetic basis for seizure, global developmental delay, and hypotonia in the DNA of the probands. We filtered the annotated variants by removing synonymous variants with combined annotation − dependent depletion (CADD) scores < 15, as well as variants with a minor allele frequency (MAF) ≥ 0.001.

**Figure 2:**
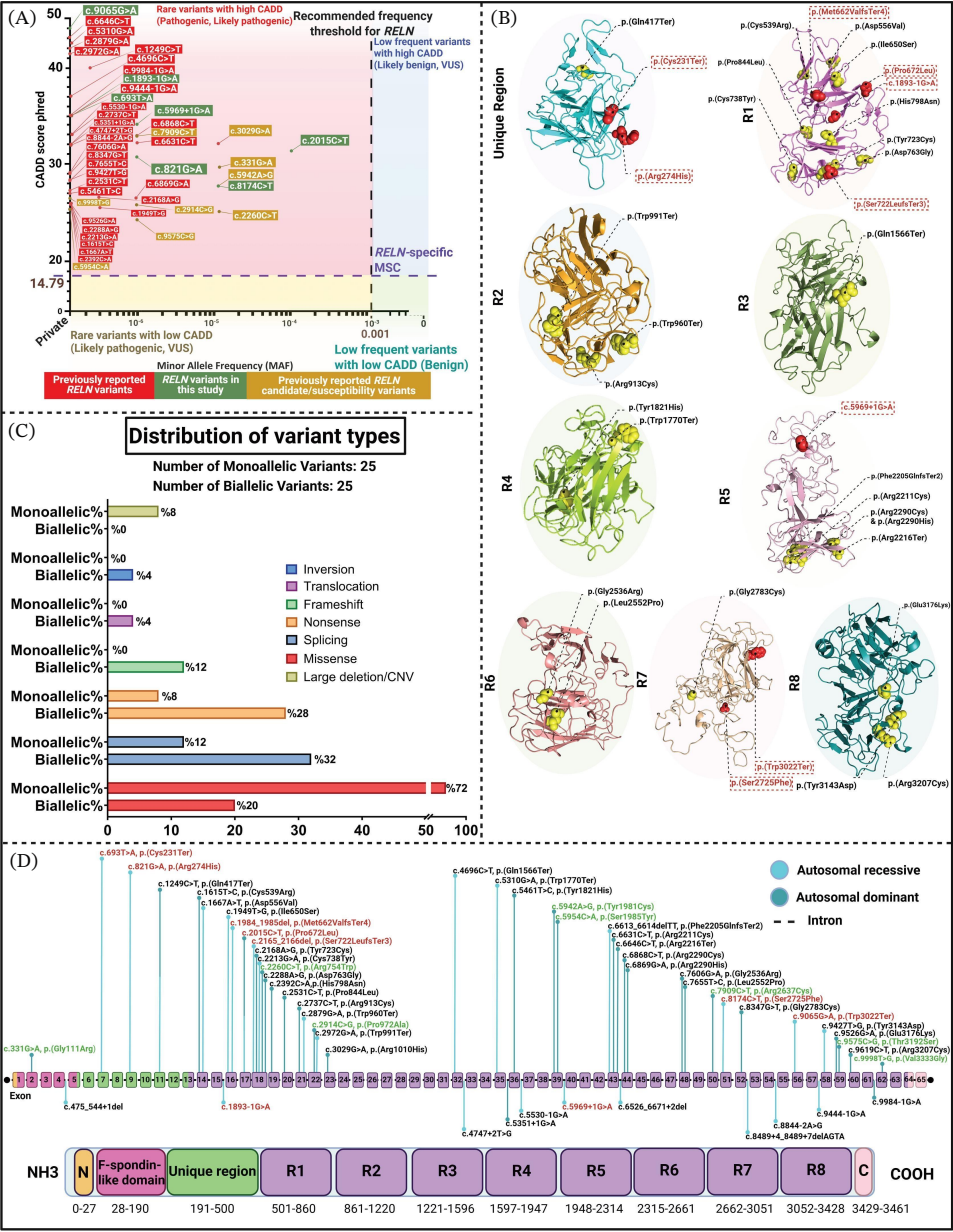
Distribution, predicted pathogenicity, and structural localization of *RELN* variants. (A) CADD scores plotted against minor allele frequency for previously reported variants included in the primary cohort (red), variants identified in the present study (green), and previously reported candidate or susceptibility variants (orange). Candidate/susceptibility variants are displayed for comparison but were excluded from all primary quantitative analyses. (B) Three‐dimensional localization of primary‐cohort variants across the unique region and Reelin repeats R1–R8. Variants identified in the present study are highlighted in red, whereas previously reported primary‐cohort variants are labeled in black; susceptibility variants are not included in the structural models. (C) Distribution of variant types across 25 monoallelic and 25 biallelic variant observations. Two variants were observed in both inheritance contexts; therefore, these observations represent 48 distinct primary‐cohort variants. (D) Linear schematic of *RELN* illustrating primary autosomal recessive variants, primary autosomal dominant variants, and candidate/susceptibility variants using distinct colors.

All 10 individuals from the eight families had biallelic *RELN* genotypes. Nine (P1–P9) were homozygous, reflecting their parents′ consanguinity, whereas the proband of Family 8 (P10) was compound heterozygous for c.821G>A, p.(Arg274His), and c.8174C>T, p.(Ser2725Phe). Although Family 8 is also consanguineous, the two variants are not identical by descent and originate from different parental lineages. Sanger sequencing revealed that c.821G>A was inherited from the unaffected heterozygous father, and c.8174C>T from the unaffected heterozygous mother. One of the two tested siblings was wild type and the other was a heterozygous carrier of the maternal c.8174C>T variant. Both were asymptomatic, consistent with AR inheritance in this family. Figure 1 shows the genotypes. Six variants were classified as pathogenic, one as likely pathogenic, and two as variants of uncertain significance. Six variants were truncating or canonical splice‐altering, likely leading to LOF, whereas three were missense variants. CADD scores for single‐nucleotide variants (SNV) far exceeded the gene‐specific mutation significance cutoff (MSC) of 14.79 [35] (see Figure 2A and Table 2). The CADD‐PHRED scores plotted against gnomAD MAF show that all SNV *RELN* variants fall above the gene‐specific MSC of 14.79 and the maximum credible population allele frequency applied in this study (0.001) for *RELN*, that is, within the “rare + high − CADD” quadrant that is compatible with pathogenicity (see Figure 2A). These variants included one previously reported variant (c.2015C>T [7, 10, 36]), four novel variants (c.1893‐1G>A, c.9065G>A, c.2165_2166del, and c.693T>A), and four variants that, although not previously reported in patients, have been described in the ClinVar (c.5969+1G>A [RCV000210930], c.1984_1985del [RCV005213821], c.821G>A [RCV001963454], and c.8174C>T [RCV001528672]). The frequencies, bioinformatics analyses, and predictions of our *RELN* variants are summarized in Table 2. The positions and consequences of these variants were mapped onto their 3D protein structures using PyMOL (see Figure 2B–D).

**Table 2:** Bioinformatics software prediction of the adverse effects of the *RELN* variants (NM_005045.4).

Genetic variant	MT ^∗^	Geno canyon	BayesDel	EIGEN	dbscSNV RF/Ada	Splice AI	DANN	CADD	Revel	MutPred2	MaxEntScan	gnomAD frequency V4.1.0 Het/Hom
c.1893‐1G>A	D^#^	D	D	D	D	Strong	D	35	NA	NA	D	NA
c.9065G>A	D	D	D	D	D	NA	D	49	NA	NA	NA	NA
c.693T>A	D	B	D	U^$^	NA	NA	D	35	NA	NA	NA	NA
c.2015C>T	D	NA	D	D	NA	NA	D	31	D	D	NA	0.000156/0
c.5969+1G>A	D	D	D	D	D	D	D	34	NA	NA	D	0.000001/0
c.821G>A	D	D	U	D	NA	NA	D	31	D	U	NA	0.000004/0
c.8174C>T	D	D	U	D	NA	NA	D	27.5	NA	D	NA	0.00003/0

Abbreviations: NA, not available; B, benign.

^∗^MutationTaster.

^#^Deleterious.

^$^Uncertain.

The allele carried by P7, c.2015C>T, p.(Pro672Leu) (rs201044262), had until now been found only in the heterozygous state, in individuals with ADLTE and in clinically unaffected carriers [7, 10, 36]. It is relatively frequent among rare disease‐associated alleles (gnomAD v4.1.0 MAF, 1.56 × 10^−4^; no homozygotes). ClinVar currently reports conflicting classifications of uncertain significance and benign, together with an additional likely benign submission. To our knowledge, P7 is the first reported homozygous individual with this variant and the full LCH phenotype. In the specific context of P7′s homozygous recessive presentation, application of PM2_Supporting, PM3_Supporting, PP3_Moderate, and PP4_Moderate supports a likely pathogenic classification in this study.

In P10, the two alleles are variants of uncertain significance and parental testing confirmed trans configuration, but neither qualifies for a higher classification. No pathogenic or likely pathogenic variants were found in any other disease‐associated gene, including those on the ACMG SF v3.3 list, across all probands [37, 38].

### 3.3. Systematic Review (Including Our Cases)

**Figure 3:**
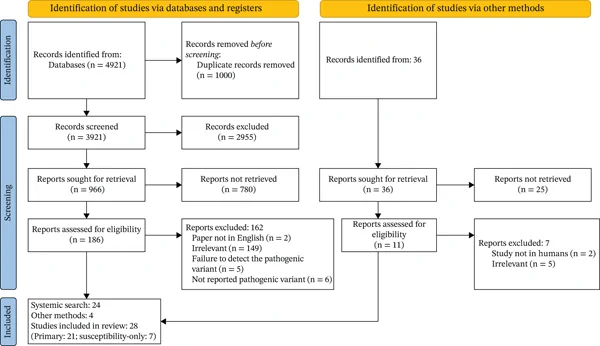
PRISMA (Preferred Reporting Items for Systematic Reviews and Meta‐Analyses) 2020 flow diagram of included and excluded articles for *RELN*‐related syndromes.

The updated search found 4921 records. We removed duplicates, screened titles and abstracts and 186 reports were assessed for eligibility resulting in 24 eligible studies. Moreover, 4 other eligible studies were identified from the 36 records identified thru other sources. A total of 28 studies were included for qualitative synthesis, with 21 contributing to the primary quantitative cohort and 7 being only included in the susceptibility dataset (see Figure 3 and Table S2).

**Figure 4:**
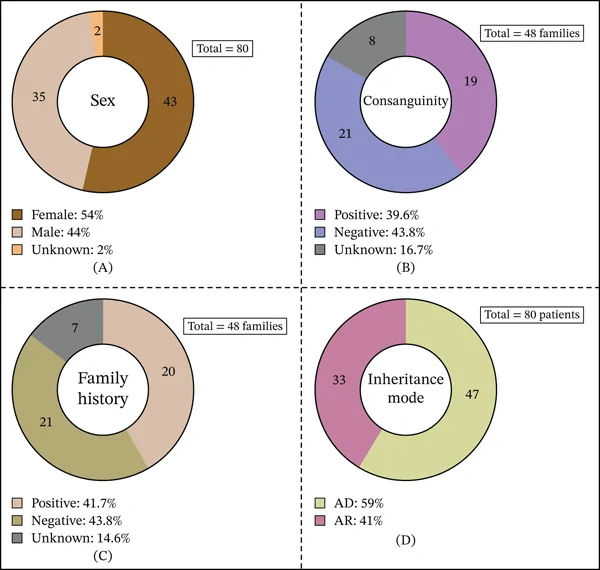
Demographic and inheritance characteristics of the primary *RELN* cohort. (A) Sex distribution among the 80 individuals. (B) Consanguinity status and (C) family history across the 48 independent kindreds. (D) Monoallelic (autosomal dominant) versus biallelic (autosomal recessive) genotypes. Values are given in the Results. Susceptibility cases were excluded from all panels.

The primary cohort included 80 individuals from 48 independent kindreds, 70 previously reported patients and 10 individuals from the current study. Females comprised 54% of the cohort (*n* = 43), males 44% (*n* = 35) and 2% were of unreported sex (*n* = 2). Consanguinity was reported in 19 families, absent in 21, and unavailable in eight of the 48 families. A positive family history was observed in 20 kindreds, whereas 21 showed no family history, and data were unavailable for seven. Forty‐seven individuals carried monoallelic variants indicative of autosomal dominant (AD) inheritance, whereas 33 had biallelic variants aligned with AR inheritance (Figure 4 and Tables S3, S4, S5) [4, 5, 9–14, 16–18, 20, 36, 39–46]. Another 16 individuals from nine kindreds were only part of the susceptibility dataset and were not included in these quantitative summaries [19, 47–52].

**Figure 5:**
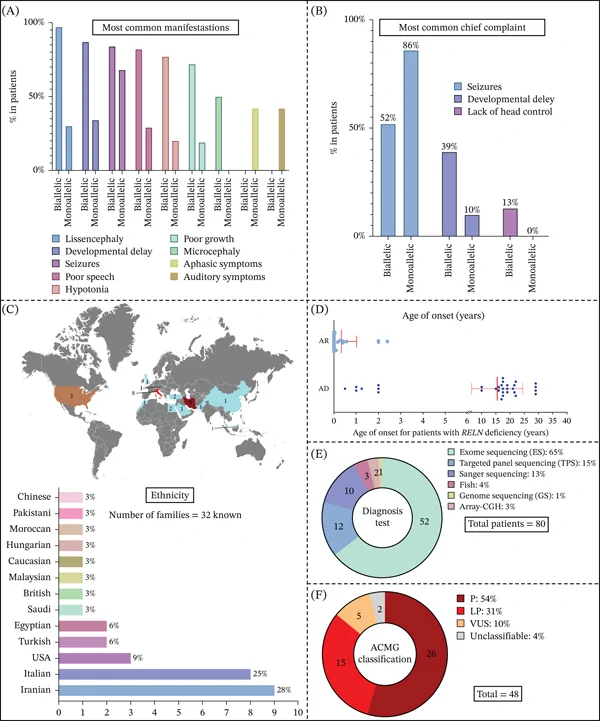
Clinical, demographic, diagnostic, and molecular characteristics of the primary *RELN* cohort. (A) Frequencies of major clinical manifestations among evaluable autosomal recessive/biallelic and autosomal dominant/monoallelic cases. (B) Most common chief presenting complaints stratified by inheritance mode. (C) Geographic and ethnic distribution of the 32 independent kindreds for which ancestry or country of origin was reported. (D) Age at onset by inheritance mode. Individual‐level values were available for 38 individuals; for 18 individuals from six ADLTE kindreds, only family‐level ranges were published, and the midpoint of each range is plotted. Points therefore span 10–29 years in the later onset group, whereas the raw reported bounds span 8–40 years (Discussion). Monoallelic individuals occurred in both onset groups. (E) Diagnostic approaches used across the 80 patients. (F) ACMG classification of the 48 distinct primary‐cohort variants. Values are given in the Results. Susceptibility variants were excluded from all panels.

Among evaluable AR cases, the most common manifestations were lissencephaly (97%), developmental delay (87%), seizures (84%), poor speech (82%), hypotonia (77%), and poor growth (72%). In the AD group, seizures were the most common manifestation (67%), followed by aphasic and auditory symptoms (42% each), developmental delay (33%), lissencephaly (30%), and poor speech (29%) (see Figure 5A). Seizures were the leading presenting symptom in 52% of AR cases and 86% of AD cases. Developmental delay was the initial concern in 39% of AR and 10% of AD cases. Lack of head control was reported as the presenting issue in 13% of AR cases, with no cases reported in AD (see Figure 5B). Ethnicity or country of origin data was available for 32 of the 48 kindreds. The largest group was Iranian families (9/32, 28%), next were Italian families (8/32, 25%), then families from the United States (3/32, 9%), and Egyptian and Turkish families (2/32, 6% each) (see Figure 5C). Out of 25 AR cases with available age‐of‐onset data, the mean age of onset was 0.35 years (SD = 0.70), and the median was 0 years. The first quartile was 0 years, and the third quartile was 0.33 years, resulting in an IQR of 0.33 years. Out of 31 AD cases, the average age at onset was 15.44 years (SD = 9.03), with a median of 17.5 years. The first and third quartiles were 10 and 21.25 years, respectively, resulting in an IQR of 11.25 years (see Figure 5D). The distribution was discontinuous, with no observations between 2.4 and 10 years. In the AD group, seven individuals experienced onset at 2 years or younger (range 0.5–2 years; all had documented cortical malformations), whereas 24 individuals had onset at 10 years or older (median 18.25 years; none with cortical malformations in evaluable cases). Therefore, when interpreting summary statistics for this group, it is important to consider this internal structure. Among 80 patients, ES was the most common diagnostic method used (52 out of 80, 65%), followed by targeted panel sequencing (12/80, 15%), Sanger sequencing (10/80, 13%), fluorescence in situ hybridization (FISH) (3/80, 4%), array‐based comparative genomic hybridization (array‐CGH) (2/80, 3%), and genome sequencing (1/80, 1%) (see Figure 5E).

The primary cohort contained 48 distinct *RELN* variants. After reassessment using a consistent ACMG framework, 26 variants (54%) were classified as pathogenic, 15 (31%) as likely pathogenic, five (10%) as variants of uncertain significance, and two balanced rearrangements (4%) are copy‐neutral and do not fit into the sequence‐variant or copy‐number categories (see Figure 5F). Among individuals, 72 out of 80 patients (90%) possessed at least one pathogenic or likely pathogenic allele. Five patients (6%) had only variants of uncertain significance, whereas three (4%) carried a balanced rearrangement. The nine candidate or susceptibility variants were summarized separately and were not included in these percentages (refer to Figure 5F and the Table S6).

Missense variants were the most common monoallelic variant observed (18 out of 25, 72%), followed by splice‐altering variants (12%), nonsense variants (8%), and large deletions/copy‐number variants (CNVs) (8%). Conversely, biallelic (two variants were seen in both zygosity contexts, so these 50 observations correspond to the 48 distinct primary‐cohort variants) observations were enriched for splice‐altering (8 out of 25, 32%) and nonsense variants (7 out of 25, 28%), followed by missense (20%), frameshift (12%), translocation (4%), and inversion (4%) variants (refer to Figure 2C,D). Four variants involved major structural changes. The two multiexon deletions within *RELN* identified by array‐CGH both have breakpoints inside the gene and remove exons that code for proteins. These were classified as pathogenic according to ClinGen/ACMG copy‐number guidelines. Both deletions were detected in a heterozygous state and are thus considered pathogenic alleles rather than direct causes of the observed phenotype. The pericentric inversion and homozygous balanced reciprocal translocation are copy‐neutral and are described separately. The most common sequence variant was c.2392C>A, p.(His798Asn), found in five individuals (6.25%). Another variant, c.8347G>T, p.(Gly2783Cys), was observed in four individuals (5%) within a single family affected by autosomal‐dominant lateral temporal epilepsy [7, 10]. This variant is not present in gnomAD v4.1.0 and impacts a highly conserved residue (phyloP100 score of 7.243). Although automated classifiers give a conservative germline call consistent with its heterozygous and reduced‐penetrance nature, it is supported by direct experimental evidence showing a LOF: Carriers exhibit decreased serum Reelin levels [10]. In cell‐based assays, the mutant protein is not secreted; instead, it is retained in the endoplasmic reticulum with blocked trafficking to the Golgi and undergoes degradation via the autophagy pathway [8]. p.(Gly2783Cys) is a confirmed LOF allele with an automated classification that depends on detection methods, illustrating the zygosity‐dependent phenotypic spectrum crucial to this study. Upon reannotation, this variant was reclassified as likely pathogenic (PS3_Moderate, PP1_Supporting, PP3_Moderate, PM2_Supporting) (see the Table S6 for the full literature review).

Alongside the primary cohort, we selected 16 individuals from 9 families carrying 9 monoallelic *RELN* variants associated with susceptibility. [19, 47–52]. All variants were heterozygous missense substitutions specific to individual families, found across exons 2–62. GnomAD frequencies were less than or equal to 8 × 10^−5^, with CADD PHRED scores ranging from 24.1 to 33.0 (median 26.5). After reassessment by ACMG/AMP, all nine remained variants of uncertain significance, scoring a maximum of 4 points. Phenotypic features included epilepsy (8/16, involving 6 families), schizophrenia (7/16), and autism (1/16). Age of onset data was available for 12/16 individuals and ranged from 14 to 39 years (median 29), with no cases presenting in infancy and none with lissencephaly, cerebellar hypoplasia, microcephaly or developmental delay. Six of the nine families were Han Chinese. These individuals were excluded from all primary cohort analyzes, and details are provided in Table S6.

## 4. Discussion

This study identifies nine biallelic *RELN* variants among 10 affected individuals from eight unrelated Iranian families with consanguinity, all exhibiting lissencephaly and cerebellar hypoplasia (LCH). Notably, the c.2015C>T, p.(Pro672Leu) variant is reported here in the homozygous form for the first time. Since the allele remains consistent, the contrast between the mild dominant and severe recessive forms appears to depend on zygosity rather than the specific variant or its predicted molecular impact, supporting the model proposed by Dazzo et al. [10] through human genetic evidence. Six variants were classified as pathogenic, one as likely pathogenic, and two as of uncertain significance. The two uncertain variants, in trans in P10, remain strong candidates due to confirmed phase, rarity, consistent computational predictions, and phenotypic specificity. Combining data with 70 previously published patients resulted in a primary cohort of 80 individuals from 48 families. This also increased the number of published biallelic cases from 23 to 33 individuals and from 14 to 22 families.

The pooled cohort follows the three‐tier architecture described earlier: biallelic LOF variants at the severe end, mainly truncating or canonical splice‐altering in the current families [4, 5, 11]. Inherited monoallelic variants lead to familial focal epilepsy without cortical malformations, showing incomplete penetrance at the milder end [10, 11, 13] and, between them, de novo monoallelic missense variants leading to dominant neuronal migration disorders [12, 53]. The clinical data follow a consistent pattern: biallelic individuals more frequently exhibit developmental delay, lissencephaly, hypotonia, poor speech, and impaired growth. Conversely, monoallelic individuals tend to exhibit focal seizures, auditory auras or aphasic symptoms with milder developmental problems, which reflects the dual functions of Reelin in embryonic neuronal migration and postnatal synaptic plasticity and network excitability [6, 15].

The age at onset was not continuously distributed. Onset data were available for 56 out of 80 individuals, with no reports of cases between 2.4 and 8 years. This created a distinction between an early‐onset group (≤ 2.4 years, *n* = 32) and a later onset group (8–40 years, *n* = 24). Individual onset data were accessible for 38 people, whereas for 18 individuals from six previously studied ADLTE kindreds, only family‐level onset ranges were published. Zygosity alone did not explain this separation: All 25 individuals with biallelic variants belonged to the early‐onset group, whereas monoallelic individuals appeared in both groups (*n* = 7 early onset; *n* = 24 later onset). The major phenotypic difference was the presence or absence of cortical malformations. All 32 early‐onset individuals had documented cortical malformations (MCD), but none of the 23 later onset individuals with evaluable neuroimaging showed evidence of MCD. Neuroimaging was mostly done with a typical 1.5‐T MRI in ADLTE families, which may be insufficient for detecting minor cortical abnormalities [7]. This trend is consistent with developmental timing: Abnormalities created during corticogenesis occur early, whereas alterations in network excitability without obvious anatomical evidence may appear later clinically. Because these cross‐sectional data do not provide direct measurements of residual Reelin activity, the association should be interpreted as descriptive rather than causal.

Monoallelic disease occurring either sporadically or through inheritance. In 47 individuals with monoallelic disease, five (10.6%) had a confirmed de novo event. Furthermore, multigenerational pedigrees displaying affected individuals along with apparently unaffected heterozygous carriers suggest incomplete penetrance and variable expressivity [11, 12, 14, 16, 19]. The penetrance of *RELN*‐related ADLTE is estimated to be around 60% [10]. This may be due to modifiers, age‐related effects, or mild phenotypes that are not recognized and which make recurrence risk estimation and predictive counseling in these families difficult. In the case of biallelic inheritance, the implications are more straightforward: consanguinity was reported in 18 of 22 AR families (82%), but only seven (32%) had a positive family history. Therefore, carrier testing and reproductive counseling should be recommended for consanguineous families regardless of family history. Kindreds reported originated from Mediterranean, Middle Eastern, European, East Asian and North American regions. Iranian and Italian families were the most frequent of the 32 kindreds with known ancestry (Figure 5C). This distribution is probably due to data collection and publication patterns rather than true prevalence.

Several proposed associations were excluded from the primary cohort. The connection between *RELN* and autism spectrum disorder or schizophrenia at the disorder level is still not fully confirmed [15, 18]. It is based on diverse variant classes found in small groups of individuals, and we have not considered either condition as a confirmed *RELN* phenotype. Although individual alleles identified in these cohorts were evaluated based on their own evidence, only the few that were classified as pathogenic or likely pathogenic were included in the primary cohort. A sensitivity analysis that excluded these alleles showed no significant change in the results (41/48, 85% vs. 37/44, 84% at the variant level; 72/80, 90% vs. 66/74, 89% at the individual level). Rare missense variants reported in myoclonus–dystonia [54] were deemed insufficient to confirm *RELN* as a sole cause of the disorder. Additionally, p.(Ser2486Gly), which was reported to segregate with ankylosing spondylitis in one family and linked to altered Reelin secretion in vitro, was not considered conclusive [55, 56], was omitted from both datasets due to the lack of replication and an explanation connecting heterozygous Reelin dysfunction to a complex inflammatory phenotype. Three heterozygous missense variants associated with genetic generalized epilepsy were supported by secretion‐assay data [50]. These were kept in the candidate/susceptibility dataset because genetic generalized epilepsy is not an established *RELN* phenotype, and the segregation evidence did not meet the threshold used in this analysis. The strongest evidence continues to support *RELN*′s key role in cortical malformations, neuronal migration disorders, and epilepsy. Variants observed in epilepsy and psychiatric groups were excluded from our causal cohort because their consistent variant of uncertain significance (VUS) classification highlights the limitations of a framework designed for high‐penetrance Mendelian variants, rather than indicating benignity. Risk alleles need a separate classification approach [57]. They therefore do not support any of the conclusions drawn here.

The neuroradiological features of LIS2 are similar to those of other genetically heterogeneous lissencephaly syndromes, and imaging alone often cannot determine the cause [30, 58]. ES accounted for 52 out of 80 variant‐detection events (65%) and was the most common method used, supporting gene‐agnostic sequencing, ES, or genome sequencing when available, as an early step in diagnosing lissencephaly, cerebellar hypoplasia, severe developmental impairment, or familial focal epilepsy [59, 60]. A molecular diagnosis streamlines the diagnostic process, helps determine recurrence risk, and supports carrier testing, reproductive counseling, and monitoring of at‐risk relatives.

Several limitations apply. The systematic component mainly used retrospective case reports and small series with inconsistent assessment and variable reporting of segregation, neuroimaging, and age at onset. Publication and ascertainment bias may have favored severe or family cases and several categories remained small after exclusion of persons with no variant data and duplicated patients across publications. When onset was reported as family level ranges, imputation was performed at the midpoint of the range; the lowest bound was 8 years, so some cases between 8 and 10 years may be hidden, but cases between 2.4 and 8 years are unlikely. For each allele, evidence for five primary‐cohort variants of uncertain significance, all missense, supports the variant, not benignity. Reduced penetrance affects segregation evidence in dominant families, and homozygosity by descent limits PM3 weight in consanguineous pedigrees. None of the three alleles in P7 and P10 have undergone functional testing, and data from other *RELN* alleles were not extrapolated. ES was performed in singleton mode, which precludes direct determination of de novo status and decreases sensitivity for structural variants, repeat expansions, noncoding variants and mosaicism. Four of 48 primary‐family variants are gross rearrangements identified cytogenetically or by microarray. A negative ES does not exclude a *RELN* cause, and further follow‐up of heterozygous carriers is required to determine the age‐related penetrance.

The data describe a wide spectrum of RELNopathies, influenced by zygosity, ranging from severe early‐onset LCH to dominant neuronal migration disorders and familial focal epilepsy. In the studied cohort, inheritance was less correlated with cortical malformations than age at onset.

## Author Contributions

S.B., M.S.A., M.G., E.E., P.J., F.P., R.K., M.M., and E.G.K. contributed to study design and/or performed experiments. S.B. contributed to writing the original draft. S.B., H.V., M.S.A., M.G., E.E., P.J., F.P., R.K., M.M., and E.G.K. contributed to the review and editing. S.B., H.R., E.A., H.V., A.S.M., M.S.A., H.G., M.G., E.E., P.J., R.K., F.P., G.S., N.C., H.R.K.K., R.S., S.S., S.N., A.E., M.L., S.S., S.N., B.B., F.H., S.A., R.G.J., M.B.T., M.H., H.M., M.M., M.M.S., and E.G.K. contributed to clinical data and material collection, followed the patient, analyzed data, and provided clinical care. S.B. contributed to supervision.

## Funding

No funding was received for this manuscript.

## Disclosure

The authors take full responsibility for the content of the publication.

## Ethics Statement

The research was performed with the approval of the Ethics Board of the Genetics Department of Shahid Chamran University of Ahvaz, Iran (code: EE/99.3.02.39641/scu.ac.ir).

## Consent

Written informed consent for publication of clinical, radiological and genetic data, including images, was obtained from the parents or legal guardians of all 10 patients.

## Conflicts of Interest

The authors declare no conflicts of interest.

## Supporting information


**Supporting Information** Additional supporting information can be found online in the Supporting Information section. **Supporting Information.** Table S1: Search strategy. Table S2: Quality assessment, country of publication of reference articles included in the systematic review. Table S3: Sex of patients with *RELN*‐related disorders. Table S4: Consanguinity distribution of families with *RELN*‐related disorders. Table S5: Family history status of families with *RELN*‐related disorders. Table S6.

## Data Availability

The data that support the findings of this study are available on request from the corresponding author. The data are not publicly available due to privacy or ethical restrictions as they contain information that could compromise patient privacy.
