# Supplementary material for: Zygosity‐Dependent Phenotypic Spectrum of RELN‐Related Disorders: 10 New Patients and Genotype–Phenotype Correlations Across 48 Kindreds
Source: Hum Mutat. 2026 Sep 17;2026:9993570. doi: 10.1155/humu/9993570 (PMC13586675; doi:10.1155/humu/9993570)
Supplement: Supplementary file 1 [file HUMU-2026-9993570-s001.zip › table/Supplementary.docx]

**Methods:**

| **Table S1: Search strategy** | |
| --- | --- |
| **Concept 1** | (“Lissencephaly 2” OR “Norman-roberts Syndrome” OR “Cobblestone Lissencephaly” OR "Norman Roberts Lissencephaly Syndrome" OR “Epilepsy, Familial Temporal Lobe, 7” OR “Familial Temporal Lobe Epilepsy 7” OR RELN[tiab] OR Reelin[tiab]) |
| **Concept 2** | (“single gene”[tiab] OR monogenic[tiab] OR genotype[mesh] OR genes[mesh] OR “pathogenic variant”[tiab] OR “sequence variant”[tiab] OR “genetic*”[tiab] OR “Mutat*”[tiab] OR “genetic cause*”[tiab] OR “Autosomal recessive”[tiab] OR “Autosomal dominant”[tiab] OR inherited[tiab] OR “exome sequencing”[mesh] OR “whole genome sequencing”[mesh] OR “genetic testing”[mesh] OR “Sanger Sequencing”[tiab] OR monoallelic[tiab] OR biallelic[tiab] OR “Panel sequencing”[tiab] OR “Hereditary”[tiab] OR RELN[tiab] OR Reelin[tiab]) |
| **Search** | concept 1” AND “concept 2” |

| **Table S2: Quality assessment, Country of publication of Reference Articles included in the systematic review** | | | | |
| --- | --- | --- | --- | --- |
| **Primary (21)** | | | | |
| **N.** | **Papers** | **Country of publication** | **Year** | **Quality assessment** |
| 1 | Afridi TUK, Fatima A, Satti HS, Akram Z, Yousafzai IK, Naeem WB, et al. Exome sequencing in four families with neurodevelopmental disorders: genotype-phenotype correlation and identification of novel disease-causing variants in VPS13B and RELN. Mol Genet Genomics. 2024;299(1):55 | Pakistan | 2024 | Good |
| 2 | Balicza P, Varga N, Bolgár B, Pentelényi K, Bencsik R, Gál A, et al. Comprehensive Analysis of Rare Variants of 101 Autism-Linked Genes in a Hungarian Cohort of Autism Spectrum Disorder Patients. Front Genet. 2019;10:434. | Hungary | 2019 | Poor |
| 3 | Balza C, Garofalo G, Cos T, Désir J, Kang X, Keymolen K, et al. A prenatal case of lissencephaly with cerebellar hypoplasia: New mutation in RELN gene. Clin Case Rep. 2021;9(12):e04882. | Belgium | 2021 | Fair |
| 4 | Bertoli-Avella AM, Beetz C, Ameziane N, Rocha ME, Guatibonza P, Pereira C, et al. Successful application of genome sequencing in a diagnostic setting: 1007 index cases from a clinically heterogeneous cohort. Eur J Hum Genet. 2021;29(1):141-53. | Saudi Arabia | 2021 | Poor |
| 5 | Bonora E, Beyer KS, Lamb JA, Parr JR, Klauck SM, Benner A, Paolucci M, Abbott A, Ragoussis I, Poustka A, Bailey AJ, Monaco AP; International Molecular Genetic Study of Autism (IMGSAC). Analysis of reelin as a candidate gene for autism. Mol Psychiatry. 2003 Oct;8(10):885-92. | UK | 2003 | Fair |
| 6 | Chang BS, Duzcan F, Kim S, Cinbis M, Aggarwal A, Apse KA, et al. The role of RELN in lissencephaly and neuropsychiatric disease. Am J Med Genet B Neuropsychiatr Genet. 2007;144b(1):58-63. | USA | 2007 | Good |
| 7 | Costain G, Lionel AC, Merico D, Forsythe P, Russell K, Lowther C, et al. Pathogenic rare copy number variants in community-based schizophrenia suggest a potential role for clinical microarrays. Hum Mol Genet. 2013;22(22):4485-501. | Canada | 2013 | Good |
| 8 | Dazzo E, Fanciulli M, Serioli E, Minervini G, Pulitano P, Binelli S, et al. Heterozygous reelin mutations cause autosomal-dominant lateral temporal epilepsy. Am J Hum Genet. 2015;96(6):992-1000. | USA | 2015 | Good |
| 9 | Di Donato N, Guerrini R, Billington CJ, Barkovich AJ, Dinkel P, Freri E, et al. Monoallelic and biallelic mutations in RELN underlie a graded series of neurodevelopmental disorders. Brain. 2022;145(9):3274-87. | USA | 2022 | Good |
| 10 | Zillhardt JL, Poirier K, Broix L, Lebrun N, Elmorjani A, Martinovic J, et al. Mosaic parental germline mutations causing recurrent forms of malformations of cortical development. Eur J Hum Genet. 2016;24(4):611-4. | France | 2016 | Good |
| 11 | Huang R, Fu F, Zhang N, Zhou H, Mei S, Han J, Deng Q, Liu H, Zhang Y, Yu Q, Pan M, Li F, Lu J, Ma C, Guo F, Chen H, Liu L, Zhao X, Tan X, Li D, Li R, Liao C. Genetic landscape and phenotypic correlations of lissencephaly: prenatal and postnatal insights. Brain Commun. 2026 Mar 6;8(2):fcag069. doi: 10.1093/braincomms/fcag069.PMID: 41853045; PMCID: PMC12993814. | China | 2026 | Fair |
| 12 | Michelucci R, Dazzo E, Volpi L, Pasini E, Riguzzi P, Minardi R, et al. Autosomal dominant lateral temporal lobe epilepsy associated with a novel reelin mutation. Epileptic Disord. 2020;22(4):443-8. | Italy | 2020 | Good |
| 13 | Neale BM, Kou Y, Liu L, Ma'ayan A, Samocha KE, Sabo A, et al. Patterns and rates of exonic de novo mutations in autism spectrum disorders. Nature. 2012;485(7397):242-5. | USA | 2012 | Fair |
| 14 | Zaki M, Shehab M, El-Aleem AA, Abdel-Salam G, Koeller HB, Ilkin Y, et al. Identification of a novel recessive RELN mutation using a homozygous balanced reciprocal translocation. Am J Med Genet A. 2007;143a(9):939-44. | Egypt | 2007 | Good |
| 15 | Fong SL, Lim KS, Salleh MZ, Teh LK. A heterozygous pathogenic RELN variant in autosomal dominant lateral temporal epilepsy. Seizure. 2025 Jul;129:22-24. doi: 10.1016/j.seizure.2025.03.015. PMID: 40174292. | Malaysia | 2025 | Fair |
| 16 | Hong SE, Shugart YY, Huang DT, Shahwan SA, Grant PE, Hourihane JO, et al. Autosomal recessive lissencephaly with cerebellar hypoplasia is associated with human RELN mutations. Nat Genet. 2000;26(1):93-6. | USA | 2000 | Good |
| 17 | Igreja L, Menezes C, Pinto PS, Freixo JP, Chorão R. Lissencephaly With Cerebellar Hypoplasia Due To a New RELN Mutation. Pediatr Neurol. 2023;149:137-40. | Portugal | 2023 | Fair |
| 18 | Iossifov I, O'Roak BJ, Sanders SJ, Ronemus M, Krumm N, Levy D, et al. The contribution of de novo coding mutations to autism spectrum disorder. Nature. 2014;515(7526):216-21. | USA | 2014 | Fair |
| 19 | Riva M, Ferreira S, Hayashi K, Saillour Y, Medvedeva VP, Honda T, et al. De novo monoallelic Reelin missense variants cause dominant neuronal migration disorders via a dominant-negative mechanism. J Clin Invest. 2024;134(16). | France | 2024 | Good |
| 20 | Sobue A, Kushima I, Nagai T, Shan W, Kohno T, Aleksic B, Aoyama Y, Mori D, Arioka Y, Kawano N, Yamamoto M, Hattori M, Nabeshima T, Yamada K, Ozaki N. Genetic and animal model analyses reveal the pathogenic role of a novel deletion of RELN in schizophrenia. Sci Rep. 2018 Aug 29;8(1):13046. | Japan | 2018 | Good |
| 21 | Valence S, Garel C, Barth M, Toutain A, Paris C, Amsallem D, et al. RELN and VLDLR mutations underlie two distinguishable clinico-radiological phenotypes. Clin Genet. 2016;90(6):545-9 | France | 2016 | Good |
| **Susceptibility-only (7)** | | | | |
| 1 | Wu X, Zhong S, Cai Y, Yang Y, Lian Y, Ding J, Wang X. Heterozygous RELN missense variants associated with genetic generalized epilepsy. Seizure. 2023;111:122-9. | China | 2023 | Good |
| 2 | Yuen RK, Thiruvahindrapuram B, Merico D, Walker S, Tammimies K, Hoang N, Chrysler C, Nalpathamkalam T, Pellecchia G, Liu Y, Gazzellone MJ, D'Abate L, Deneault E, Howe JL, Liu RS, Thompson A, Zarrei M, Uddin M, Marshall CR, Ring RH, Zwaigenbaum L, Ray PN, Weksberg R, Carter MT, Fernandez BA, Roberts W, Szatmari P, Scherer SW. Whole-genome sequencing of quartet families with autism spectrum disorder. Nat Med. 2015 Feb;21(2):185-91. | Canada | 2015 | Good |
| 3 | Zhou Z, Hu Z, Zhang L, Hu Z, Liu H, Liu Z, et al. Identification of RELN variation p.Thr3192Ser in a Chinese family with schizophrenia. Sci Rep. 2016;6:24327. | China | 2016 | Fair |
| 4 | Fang XQ, Zhang RR, Liu XW. Heterozygous missense mutation of the RELN gene is one of the causes of epilepsy. Neurol Res. 2022;44(3):262-7. | China | 2022 | Fair |
| 5 | Fromer M, Pocklington AJ, Kavanagh DH, Williams HJ, Dwyer S, Gormley P, et al. De novo mutations in schizophrenia implicate synaptic networks. Nature. 2014;506(7487):179-84. | USA | 2014 | Fair |
| 6 | Li Z, Wang F, He Z, Guo Q, Zhang J, Liu S. RELN gene-related drug-resistant epilepsy with periventricular nodular heterotopia treated with radiofrequency thermocoagulation: a case report. Front Neurol. 2024 Mar 27;15:1366776. doi: 10.3389/fneur.2024.1366776.PMID: 38601336; PMCID: PMC11004351. | China | 2024 | Fair |
| 7 | Salman B, Kesim Y, Şirin NG, Süsgün S, Uzun GA, İşeri SU, Bebek N, Baykan B. Novel Genetic Insights into Lateral Temporal Lobe Epilepsy: Findings from Whole Exome Sequencing. Noro Psikiyatr Ars. 2026 Feb 19;63:180-186. doi: 10.29399/npa.28894. PMID: 41777496; PMCID: PMC12951495. | Turkey | 2026 | Fair |

| Table S3: Sex of patients with *RELN*-Related Disorders | | |
| --- | --- | --- |
| Sex | N | % |
| Male | 35 | 44% |
| Female | **43** | **54%** |
| Unknown | 2 | 2% |
| All | 80 | 100% |

| Table S4: Consanguinity distribution of families with *RELN*-Related Disorders | | |
| --- | --- | --- |
| Consanguinity | N | % |
| + | 19 | 40% |
| - | **21** | **44%** |
| Unknown | **8** | **16%** |
| All | 48 | 100% |

| Table S5: Family History status of families with *RELN*-Related Disorders | | |
| --- | --- | --- |
| Family History | N | % |
| + | 20 | 42% |
| - | **21** | **44%** |
| Unknown | 7 | 14% |
| All | 48 | 100% |
